# Industry Payments for Vibegron and Prescribing Patterns Among Urologic Clinicians

**DOI:** 10.1001/jamahealthforum.2023.4020

**Published:** 2023-12-21

**Authors:** Kayla Polcari, Max J. Hyman, Ted A. Skolarus, Anne E. Sales, David O. Meltzer, Parth K. Modi

**Affiliations:** 1 Center for Health and the Social Sciences, University of Chicago, Chicago, Illinois; 2 Section of Urology, Department of Surgery, University of Chicago, Chicago, Illinois; 3 Center for Clinical Management Research, Veterans Affairs Ann Arbor Healthcare System, Ann Arbor, Michigan; 4 Department of Family and Community Medicine, University of Missouri, Columbia; 5 Sinclair School of Nursing, University of Missouri School of Medicine, Columbia; 6 Department of Medicine and Economics, Harris School of Public Policy Studies, University of Chicago, Chicago, Illinois; 7 Department of Surgery, University of Chicago, Chicago, Illinois

## Abstract

This cross-sectional study compares the prescribing practices among urologists and advanced practice clinicians who received vs did not receive payment from drug manufacturers.

## Introduction

Urologists who receive pharmaceutical industry payments often prescribe more expensive marketed medications.^1^ However, the association between payments and prescribing among advanced practice clinicians (APCs) in urology is unknown, despite APCs receiving $119 million from the industry in 2021.^2,3^ Vibegron is the newest, highest-cost overactive bladder (OAB) medication approved by the US Food and Drug Administration.^4,5^ We measured the association between industry payments and prescribing patterns for vibegron by urologists and APCs during its first year on the market.

## Methods

We performed a cross-sectional study of urologists and APCs (physician assistants and nurse practitioners) in single-specialty urology practices, defined as those in which most physicians were urologists according to the 2020 Medicare Data on Provider Practice and Specialty file. Clinicians who prescribed at least 11 claims of any OAB medication in the 2021 Medicare Part D Public Use File were included (eTable in Supplement 1). We used Open Payments data to calculate the value and number of payments for vibegron in 2021. The University of Chicago Institutional Review Board deemed this study exempt from ethics review and informed consent requirement because publicly available data were used. We followed the STROBE reporting guideline.

Primary outcomes were a clinician prescribing vibegron, percentage of a clinician’s OAB prescriptions for vibegron, and percentage of a clinician’s OAB medication days’ supply for vibegron. Descriptive statistics were performed with χ^2^ tests; 2-sample, 2-sided *t* tests; and Mann-Whitney tests. Multivariable logistic and linear regressions tested the association between industry payment and prescribing. Covariates included clinician type, sex, age, and practice size and patient demographics (age, risk score, race and ethnicity [collected to account for differences in patient populations that might alter payments or prescribing], and low-income subsidy). Interaction term was included to determine whether the association between payment and prescribing differed by clinician type.

*P* < .05 indicated statistical significance. Data analysis was performed with SAS 9.4 (SAS Institute Inc) and Stata 17 (StataCorp LLC).

## Results

We included 4616 clinicians (952 females [20.6%], 3664 males [79.4%]; mean [SD] age, 52.5 [12.7] years): 3763 urologists and 853 APCs (Table 1). Industry payments for vibegron were received by 30.8% (1158 of 3763) of urologists and 36.0% (307 of 853) of APCs in 2021. Median (IQR) total value of payments was $26.57 ($16.90-$43.37), and median (IQR) number of payments was 1 (1-2). Clinicians who received payments were often male, were younger, worked in larger practices, treated more non-Hispanic White patients, and prescribed more vibegron than those who received no payments.

**Table 1.** Characteristics of Clinicians Who Received or Did Not Receive Industry Payments for Vibegron in 2021

Characteristic	Clinicians		*P* value
	Received industry payment (n = 1465)	Received no industry payment (n = 3151)	
Clinician sex, No. (%)			
Female	349 (23.8)	603 (19.1)	<.001
Male	1116 (76.2)	2548 (80.9)	
Clinician age, mean (SD), y	50.9 (11.5)	53.2 (13.2)	<.001
Clinician type, No. (%)			
Urologist	1158 (79)	2605 (82.7)	.003
APC	307 (21)	546 (17.3)	
Age of Medicare Part D beneficiaries, mean (SD), y	74.4 (1.5)	74.3 (2.6)	.10
HCC risk score of Medicare Part D beneficiaries, mean (SD)	1.32 (0.18)	1.35 (0.21)	<.001
Percentage of Medicare Part D beneficiaries with non-Hispanic White race and ethnicity, mean (SD)^a^	77.2 (18.4)	75.4 (23.1)	.005
Percentage of claims for Medicare Part D beneficiaries with low-income subsidy, mean (SD)	20.6 (14.5)	20.7 (15.8)	.93
No. of physicians in clinician practice, median (IQR)	15 (4-38)	9 (2-25)	<.001
Vibegron prescribers, No. (%)	290 (19.8)	197 (6.3)	<.001
Vibegron prescriptions, mean (SD)	5.3 (14.6)	1.4 (7.1)	<.001
Vibegron days’ supply, mean (SD)	211.1 (595.5)	57.8 (284.5)	<.001
Percentage of OAB prescriptions for vibegron, mean (SD)	1.3 (3.7)	0.4 (2.1)	<.001
Percentage of OAB medication days’ supply for vibegron, mean (SD)	1.1 (3)	0.3 (1.8)	<.001
Total value of industry payments for vibegron, median (IQR), $	26.57 (16.90-43.37)	NA	NA
Total No. of industry payments for vibegron, median (IQR)	1 (1-2)	NA	NA

Abbreviations: APC, advanced practice clinician; HCC, hierarchical condition category; NA, not applicable; OAB, overactive bladder.

^a^ Race and ethnicity data were obtained from the Medicare Part D Prescribers File and included American Indian or Alaska Native, Asian or Pacific Islander, Hispanic, non-Hispanic Black or African American, non-Hispanic White, and Other. The table included only the non-Hispanic White category because it accounted for most beneficiaries treated by the clinicians.

In adjusted analyses, clinicians who received any payment for vibegron were more likely to prescribe the medication than those who received no payment (odds ratio [OR], 3.61; 95% CI, 2.89-4.51; *P* < .001) (Table 2). Receipt of any payment was associated with an increase of 0.93% (95% CI, 0.74%-1.13%) in vibegron prescriptions as a percentage of total OAB prescriptions. Vibegron prescriptions as a percentage of total OAB claims was 1.30% (95% CI, 1.18%-1.46%) for those receiving payments vs 0.42% (95% CI, 0.33%-0.52%) for those receiving no payments. Receipt of any payment was associated with an increase of 0.77% (95% CI, 0.62%-0.93%; *P* < .001) in days’ supply of vibegron prescriptions as a percentage of total days supply of OAB medications. The association of payments with prescribing did not differ by clinician type, although APCs were less likely to prescribe vibegron (OR, 0.37; 95% CI, 0.23-0.59; *P* < .001).

**Table 2.** Association Between Prescribing Vibegron and Any Industry Payment for Vibegron Among Clinicians in 2021 (N = 4616)

Variable	Any vibegron prescriptions		Percentage of OAB prescriptions for vibegron		Percentage of OAB medication days’ supply for vibegron	
	Logistic regression		Linear regression		Linear regression	
	OR (95% CI)	*P* value	Linear coefficient (95% CI)	*P* value	Linear coefficient (95% CI)	*P* value
Clinician type						
Urologist	1 [Reference]		1 [Reference]		1 [Reference]	
APC	0.37 (0.23 to 0.59)	<.001	−0.51 (−0.83 to −0.19)	.002	−0.41 (−0.67 to −0.15)	.002
Any industry payment for vibegron	3.61 (2.89 to 4.51)	<.001	0.93 (0.74 to 1.13)	<.001	0.77 (0.62 to 0.93)	<.001
Clinician type × any industry payment for vibegron						
Urologist	1 [Reference]		1 [Reference]		1 [Reference]	
APC	1.06 (0.64 to 1.78)	.81	−0.19 (−0.61 to 0.24)	.39	−0.18 (−0.53 to 0.17)	.31
Clinician sex						
Female	1 [Reference]		1 [Reference]		1 [Reference]	
Male	0.47 (0.35 to 0.63)	<.001	−0.49 (−0.76 to −0.23)	<.001	−0.40 (−0.62 to −0.18)	<.001
Clinician age	0.97 (0.96 to 0.98)	<.001	−0.017 (−0.024. −0.01)	<.001	−0.013 (−0.19 to −0.007)	<.001
Mean age of Medicare Part D beneficiaries	1.26 (1.18 to 1.34)	<.001	0.12 (0.08 to 0.15)	<.001	0.09 (0.06 to 0.13)	<.001
Mean HCC risk score of Medicare Part D beneficiaries	0.77 (0.43 to 1.37)	.37	−0.20 (−0.63 to 0.24)	.38	−0.16 (−0.52 to 0.20)	.39
Percentage of Medicare Part D beneficiaries with non-Hispanic White race and ethnicity	1.01 (1.00 to 1.01)	.08	−0.003 (−0.007 to 0.001)	.15	−0.002 (−0.005 to 0.001)	.26
Percentage of claims for Medicare Part D beneficiaries with low-income subsidy	1.03 (1.02 to 1.04)	<.001	0.012 (0.006 to 0.018)	<.001	0.012 (0.007 to 0.017)	<.001
No. of physicians in clinician practice	1.00 (1.00 to 1.00)	.56	−0.002 (−0.005 to 0.001)	.23	−0.001 (−0.004 to 0.001)	.22

Abbreviations: APC, advanced practice clinician; HCC, hierarchical condition category; OAB, overactive bladder; OR, odds ratio.

## Discussion

Urologists and APCs who received industry payments for vibegron were more likely to prescribe it and prescribed it more often than those who did not receive payments. While this study cannot prove causality, it adds APC data to the literature and suggests that payments affect prescribing behavior similarly for both physicians and APCs. Generalizability of this study may be limited as it included only clinicians from single-specialty practices and only prescriptions for Medicare beneficiaries, although OAB prescription is most common in people older than 65 years.^6^ Nonetheless, these findings are novel for APC prescribers and consistent with existing data for physicians. Clinicians should be educated on the potential implications of financial relationships with the pharmaceutical industry.

## Notes

Supplement 1. **eTable.** Generic Names and National Drug Codes for Overactive Bladder Medications

Supplement 2.
Data Sharing Statement
