# Supplementary material for: Industry Payments for Vibegron and Prescribing Patterns Among Urologic Clinicians
Source: JAMA Health Forum. 2023 Dec 21;4(12):e234020. doi: 10.1001/jamahealthforum.2023.4020 (PMC10739068; doi:10.1001/jamahealthforum.2023.4020)
Supplement: Supplement 1. — eTable. Generic Names and National Drug Codes for Overactive Bladder Medications [file jamahealthforum-e234020-s001.pdf]

Supplementary Online Content

Polcari K, Hyman MJ, Skolarus TA, et al. Industry payments for vibegron and prescribing patterns among urologic clinicians. *JAMA Health Forum.* Published online December 21, 2023. doi:10.1001/jamahealthforum.2023.4020

eTable. Generic Names and National Drug Codes for Overactive Bladder Medications

This supplementary material has been provided by the authors to give readers additional information about their work.

© 2023 Polcari K et al. *JAMA Health Forum.*

eTable: Generic Names and National Drug Codes for Overactive Bladder Medications

Medication	Generic Name	National Drug Codes
Darifenacin	Darifenacin Hydrobromide	13668-202, 13668-203, 16571-767, 16571-768, 33342-276, 33342-277, 46708-223, 46708-224, 52605-067, 52605-068, 62332-223, 62332-224, 65862-861, 65862-862, 69097-431, 69097-432, 70700-182, 70700-183, 70754-161, 70754-162, 71205-900, 71205-901
Fesoterodine	Fesoterodine Fumarate	0069-0242, 0069-0244, 43598-247, 43598-248, 51407-661, 51407-662, 63539-183, 63539-242, 65862-766, 65862-767, 67877-064, 67877-068, 68382-479, 68382-480, 70771-1168, 70771-1169
Mirabegron	Mirabegron	0469-2601, 0469-2602, 0469-5020, 55154-8712, 55154-8713, 70518-2435, 70518-3158
Oxybutynin	Oxybutynin Chloride	0603-4975, 0615-3512, 0615-8219, 0615-8220, 0615-8428, 0832-0038, 0904-2821, 0904-6570, 0904-7027, 10702-201, 14539-653, 16729-317, 16729-318, 16729-319, 17856-0092, 23155-810, 27241-155, 27241-156, 27241-157, 42291-633, 42291-634, 42291-635, 42291-914, 43063-790, 43063-851, 43063-931, 43353-282, 43353-284, 43353-285, 43353-322, 43353-367, 43353-978, 50090-0318, 50090-2049, 50090-4192, 50090-4307, 50090-4622, 50090-4658, 50090-4728, 50090-5352, 50090-5353, 50090-5822, 50111-456, 50268-627, 50268-628, 50268-629, 50458-805, 50458-810, 51407-099, 52817-260, 53808-1122, 54838-510, 55154-0657, 60432-092, 60687-670, 62175-270, 62175-271, 62175-272, 63187-749, 63629-1354, 63739-548, 64380-162, 64980-209, 64980-210, 64980-211, 64980-431, 65162-371, 65162-372, 65162-373, 66267-642, 67296-1175, 67544-569, 68071-1875, 68084-400, 68084-480, 68084-610, 68382-255, 68382-256, 68382-257, 68788-7785, 68788-8141, 69315-182, 69452-119, 69452-120, 69452-121, 70518-1941, 70518-2388, 70518-2640, 70518-2688, 70518-2710, 70518-2904, 70518-3032, 70518-3473, 70771-1086, 70771-1087, 70771-1088, 70934-909, 70954-005, 71205-667, 71335-0519, 71335-0610, 71335-0669, 71335-0974, 71335-1014, 71335-1160, 71335-1494, 71335-1690, 71335-2121, 71610-277, 71610-347, 71610-394, 71930-051, 72189-130, 72189-131, 72189-132, 72789-049, 72888-030, 72888-031, 72888-032
Solifenacin	Solifenacin Succinate	0591-3795, 0591-3796, 27241-037, 27241-038, 29300-328, 29300-329, 31722-027, 31722-028, 35561-285, 35561-286, 42291-739, 42291-740, 46708-192, 46708-193, 50228-427, 50228-428, 51248-150, 51248-151, 51248-250, 51407-227, 51407-228, 51407-471, 51407-472, 60505-4702, 60505-4703, 62332-192, 62332-193, 63629-8387, 63629-8532, 63629-8867, 63629-8868, 63629-8869, 63629-8870, 67184-0517, 67877-527, 67877-528, 68382-987, 68382-988, 68462-386, 68462-387, 69097-259, 69097-261, 69238-1308, 69238-1309, 69367-239, 69367-240, 69367-295, 69367-296, 69539-050, 69539-051, 69844-047, 69844-048, 70518-3156, 70771-1604, 70771-1605, 71205-539, 71205-540, 71205-559, 71205-561, 71205-580, 71205-583, 71205-933, 71205-934, 71205-986, 71205-987, 71335-1729, 72205-020, 72205-021, 72606-009, 72606-010
Tolterodine	Tolterodine Tartrate	0009-4541, 0009-4544, 0009-5190, 0009-5191, 0093-0010, 0093-0018, 0093-7163, 0093-7164, 0904-6592, 0904-6593, 10135-706, 10135-707, 13668-189, 13668-190, 16571-126, 16571-127, 27241-191, 27241-192, 29300-239, 29300-

© 2023 Polcari K et al. *JAMA Health Forum.*

		240, 31722-607, 31722-608, 31722-805, 31722-806, 33342-097, 33342-098, 43975-322, 43975-323, 50090-3475, 50090-5995, 59762-0047, 59762-0048, 59762-0170, 59762-0800, 60687-319, 60687-330, 70436-160, 70436-161, 70518-1416, 70518-2794, 71205-319, 73152-026, 73152-027
Trospium	Trospium Chloride	0574-0118, 0574-0145, 0591-3636, 0904-7059, 23155-530, 35573-446, 50090-5994, 60429-098, 63629-8457, 63629-8458, 63629-9279, 68001-427, 68462-461, 69097-912, 70010-027, 70436-174, 71205-917, 76282-336
Vibegron	Vibegron	73336-075

Notes: Generic names were used to identify medications in the Medicare Part D Public Use files. National Drug Codes were used to identify medications in the Open Payments Program data.

© 2023 Polcari K et al. *JAMA Health Forum.*
