# Supplementary material for: Industry Payments for Vibegron and Prescribing Patterns Among Urologic Clinicians
Source: JAMA Health Forum. 2023 Dec 21;4(12):e234020. doi: 10.1001/jamahealthforum.2023.4020 (PMC10739068; doi:10.1001/jamahealthforum.2023.4020)
Supplement: Supplement 2. — Data Sharing Statement [file jamahealthforum-e234020-s002.pdf]

Data Sharing Statement

Polcari. Industry Payments for Vibegron and Prescribing Patterns Among Urologic Clinicians. *JAMA Health Forum*. Published December 21, 2023. doi:10.1001/jamahealthforum.2023.4020

Data
Data available: No

Additional Information
Explanation for why data not available: The publicly available Open Payments data and Medicare data we used are under Data Use Agreements that prevent us from sharing the data.
